# Global respiratory syncytial virus-associated mortality in young children (RSV GOLD): a retrospective case series

**DOI:** 10.1016/S2214-109X(17)30344-3

**Published:** 2017-09-11

**Authors:** Nienke M Scheltema, Angela Gentile, Florencia Lucion, D James Nokes, Patrick K Munywoki, Shabir A Madhi, Michelle J Groome, Cheryl Cohen, Jocelyn Moyes, Kentigern Thorburn, Somsak Thamthitiwat, Hitoshi Oshitani, Socorro P Lupisan, Aubree Gordon, José F Sánchez, Katherine L O'Brien, PERCH Study Group, Bradford D Gessner, Agustinus Sutanto, Asuncion Mejias, Octavio Ramilo, Najwa Khuri-Bulos, Natasha Halasa, Fernanda de-Paris, Márcia Rosane Pires, Michael C Spaeder, Bosco A Paes, Eric A F Simões, Ting F Leung, Maria Tereza da Costa Oliveira, Carla Cecília de Freitas Lázaro Emediato, Quique Bassat, Warwick Butt, Hsin Chi, Uzma Bashir Aamir, Asad Ali, Marilla G Lucero, Rodrigo A Fasce, Olga Lopez, Barbara A Rath, Fernando P Polack, Jesse Papenburg, Srđan Roglić, Hisato Ito, Edward A Goka, Diederick E Grobbee, Harish Nair, Louis J Bont

**Affiliations:** a Department of Paediatric Infectious Diseases and Immunology, Wilhelmina Children's Hospital, University Medical Centre Utrecht, Utrecht, Netherlands; b ReSViNET Respiratory Syncytial Virus Network, Utrecht, Netherlands; c Department of Epidemiology, Ricardo Gutiérrez Children's Hospital, Buenos Aires, Argentina; d Kenya Medical Research Institute, Wellcome Trust Research Programme, Centre for Geographic Medicine Research—Coast, Kilifi, Kenya; e School of Life Sciences, University of Warwick, Coventry, UK; f Department of Nursing Sciences, Pwani University, Kilifi, Kenya; g Medical Research Council: Respiratory and Meningeal Pathogens Research Unit and Department of Science and Technology/National Research Foundation: Vaccine Preventable Diseases, University of the Witwatersrand, Johannesburg, South Africa; h School of Public Health, University of the Witwatersrand, Johannesburg, South Africa; i Centre for Respiratory Disease and Meningitis, National Institute for Communicable Diseases, Johannesburg, South Africa; j Department of Paediatric Intensive Care, Alder Hey Children's Hospital, Liverpool, UK; k Division of Global Health Protection, Thailand Ministry of Public Health—US Centers for Disease Control and Prevention Collaboration, Nonthaburi, Thailand; l Department of Virology, Tohoku University Graduate School of Medicine, Aoba-ku, Sendai, Miyagi, Japan; m Research Institute for Tropical Medicine, Alabang Muntinlupa City, Metro Manila Philippines; n Department of Epidemiology, School of Public Health, University of Michigan, MI, USA; o Department of Medicine, Hospital Infantil Manuel de Jesus Rivera, Managua, Nicaragua; p International Vaccine Access Center, Johns Hopkins Bloomberg School of Public Health, Baltimore, MD, USA; q Agence de Medecine Preventive, Paris, France; r West Nusa Tenggara Provincial Government, Lombok, Indonesia; s Department of Pediatrics, Division of Infectious Diseases, Ohio State University, Columbus, OH, USA; t Center for Vaccines and Immunity at Nationwide Children's Hospital, Ohio State University, Columbus, OH, USA; u Department of Pediatrics, University of Jordan, Aljubeiha, Amman, Jordan; v Vanderbilt University Medical Center, Nashville, TN, USA; w Molecular Biology Laboratory, Hospital de Clínicas de Porto Alegre, Bairro Santa Cecília, Porto Alegre, Brazil; x Infection Control Commission, Hospital de Clínicas de Porto Alegre, Bairro Santa Cecília, Porto Alegre, Brazil; y Division of Pediatric Critical Care, University of Virginia School of Medicine, Charlottesville, VA, USA; z Neonatal Division, Department of Pediatrics, McMaster University, Hamilton, ON, Canada; aa Department of Pediatrics and Center for Global Health, University of Colorado, Aurora, CO, USA; ab Department of Paediatrics, Faculty of Medicine and Chinese University of Hong Kong-University Medical Center Utrecht Joint Research Laboratory of Respiratory Virus and Immunobiology, Chinese University of Hong Kong, Shatin, New Territories, Hong Kong Special Administrative Region, China; ac Health Secretariat of the City of Belo Horizonte, Belo Horizonte, Brazil; ad ISGlobal, Barcelona Centre for International Health Research, Hospital Clínic-Universitat de Barcelona, Barcelona, Spain; ae ICREA, Catalan Institution for Research and Advanced Studies, Barcelona, Spain; af Department of Pediatrics, Hospital Sant Joan de Déu, Barcelona, Spain; ag Centro de Investigação em Saúde de Manhiça, Maputo, Mozambique; ah Faculty of Medicine, Universidad Europea de Madrid, Madrid, Spain; ai Department of Intensive Care, Royal Children's Hospital, Melbourne, VIC, Australia; aj Department of Paediatrics, University of Melbourne, Melbourne, VIC, Australia; ak Murdoch Children's Research Institute, Parkville, VIC, Australia; al Department of Pediatric Infectious Disease, MacKay Children's Hospital, Taipei, Taiwan; am Department of Virology, National Institute of Health, Islamabad, Pakistan; an Department of Paediatrics and Child Health, Aga Khan University, Karachi, Pakistan; ao Public Health Institute, Ñuñoa, Santiago, Chile; ap Hospital Dr. Ernesto Torres Galdames, Iquique, Chile; aq Vienna Vaccine Safety Initiative, Berlin, Germany; ar University of Nottingham School of Medicine, Nottingham, UK; as Fundacion Infant, Buenos Aires, Argentina; at Department of Microbiology, Division of Pediatric Infectious Diseases, McGill University Health Centre, Montreal, QC, Canada; au Department of Paediatric Infectious Diseases, University Hospital for Infectious Diseases, Zagreb, Croatia; av Department of Pediatrics, Nantan General Hospital, Ueno, Yagichoyagi, Nantan-shi, Kyoto, Japan; aw School of Health and Related Research, University of Sheffield, Sheffield, UK; ax Julius Global Health, Julius Center for Health Sciences and Primary Care, University Medical Center Utrecht, Utrecht, Netherlands; ay Julius Clinical Science, Zeist, Netherlands; az Centre for Global Health Research, Usher Institute of Population Health Sciences and Informatics, University of Edinburgh, Edinburgh, UK

## Abstract

**Background:**

Respiratory syncytial virus (RSV) infection is an important cause of pneumonia mortality in young children. However, clinical data for fatal RSV infection are scarce. We aimed to identify clinical and socioeconomic characteristics of children aged younger than 5 years with RSV-related mortality using individual patient data.

**Methods:**

In this retrospective case series, we developed an online questionnaire to obtain individual patient data for clinical and socioeconomic characteristics of children aged younger than 5 years who died with community-acquired RSV infection between Jan 1, 1995, and Oct 31, 2015, through leading research groups for child pneumonia identified through a comprehensive literature search and existing research networks. For the literature search, we searched PubMed for articles published up to Feb 3, 2015, using the key terms “RSV”, “respiratory syncytial virus”, or “respiratory syncytial viral” combined with “mortality”, “fatality”, “death”, “died”, “deaths”, or “CFR” for articles published in English. We invited researchers and clinicians identified to participate between Nov 1, 2014, and Oct 31, 2015. We calculated descriptive statistics for all variables.

**Findings:**

We studied 358 children with RSV-related in-hospital death from 23 countries across the world, with data contributed from 31 research groups. 117 (33%) children were from low-income or lower middle-income countries, 77 (22%) were from upper middle-income countries, and 164 (46%) were from high-income countries. 190 (53%) were male. Data for comorbidities were missing for some children in low-income and middle-income countries. Available data showed that comorbidities were present in at least 33 (28%) children from low-income or lower middle-income countries, 36 (47%) from upper middle-income countries, and 114 (70%) from high-income countries. Median age for RSV-related deaths was 5·0 months (IQR 2·3–11·0) in low-income or lower middle-income countries, 4·0 years (2·0–10·0) in upper middle-income countries, and 7·0 years (3·6–16·8) in high-income countries.

**Interpretation:**

This study is the first large case series of children who died with community-acquired RSV infection. A substantial proportion of children with RSV-related death had comorbidities. Our results show that perinatal immunisation strategies for children aged younger than 6 months could have a substantial impact on RSV-related child mortality in low-income and middle-income countries.

**Funding:**

Bill & Melinda Gates Foundation.

## Introduction

Respiratory syncytial virus (RSV) infection is the primary pathogen identified in children with acute lower respiratory tract infection during the first year of life.^1^, ^2^, ^3^, ^4^ RSV-related acute lower respiratory tract infection is an important cause of death in young children (aged younger than 5 years); approximately 48 000–74 500 children in this age group died in hospital with the condition in 2015.^5^ About 99% of RSV-related childhood mortality occurs in developing countries.^5^ Although RSV-related mortality in children poses an important global health problem, clinical data for global RSV-related mortality are scarce. Data suggest that most RSV-related childhood mortality occurs during the first year of life.^6^, ^7^, ^8^ Although the case fatality rate is highest in children with underlying conditions, such as congenital heart disease, chronic lung disease, Down's syndrome, or premature birth,^7^, ^8^, ^9^, ^10^, ^11^, ^12^, ^13^ most cases of life-threatening RSV infection occur among previously healthy children.^14^, ^15^, ^16^ This finding suggests that in settings without intensive care facilities, otherwise healthy children could also be at risk of dying from RSV infection. A study from Argentina^16^ reported that poor access to intensive care was associated with RSV-related death.

RSV-related mortality in young children has primarily been described sporadically in studies^10^, ^11^, ^14^, ^17^ from intensive care units in high-income or middle-income countries. To date, the largest case series^11^ of 35 RSV-related deaths was reported from an intensive care unit in the UK, with numbers smaller than 35 having been reported in other studies.^2^, ^16^, ^18^, ^19^ In 2015, the WHO's Product Development for Vaccines Advisory Committee identified RSV as “a pathogen for which there is major vaccine pipeline activity”, with a vaccine likely to be available in the next 5–10 years.^20^

**Research in context**

**Evidence before this study**

Respiratory syncytial virus (RSV) infection is a leading cause of global acute lower respiratory tract infection in young children. It was associated with 48 000–74 500 in-hospital deaths in children aged younger than 5 years in 2015, with 99% of these deaths occurring in developing countries. However, individual patient data for RSV-related deaths are scarce. We searched PubMed for articles published in English up to July 7, 2017, using search terms related to RSV infection, pneumonia, and childhood mortality. We found case series of RSV-related child deaths that reported 35 cases or fewer.

**Added value of this study**

We did, to our knowledge, the first case series of children who died with RSV infection to define clinical and socioeconomic characteristics of RSV-related mortality. We searched the literature using PubMed for “RSV”, “respiratory syncytial virus”, or “respiratory syncytial viral” combined with “mortality”, “fatality”, “death”, “died”, “deaths”, or “CFR” for articles published up to Feb 3, 2015, in English, to identify research groups with relevant cases and obtained additional cases through existing research networks. We report on 358 in-hospital deaths with laboratory-confirmed RSV infection from 23 countries across the world. A substantial proportion of in-hospital RSV-related deaths occurred in children with pre-existing comorbidities. Most children in low-income and middle-income countries were aged younger than 6 months at the time of death.

**Implications of all the available evidence**

This study is the first case series of children who died with RSV infection in hospital, giving insight into the clinical and socioeconomic background of children with RSV-related death. Young age at death supports the concept that maternal vaccination against RSV infection could be an effective strategy to prevent RSV-related childhood mortality.

Two broad approaches to RSV immunisation are being considered in young children: maternal immunisation for children aged younger than 6 months and paediatric vaccines for children aged older than 6 months. A good understanding of the age distribution of RSV-related deaths is likely to assist in development of an evidence base to inform vaccine policy, particularly in low-income and middle-income settings.^21^

Previous reports of RSV-related mortality in young children described studies done in one centre, region, or country. None of them were large enough to draw robust conclusions on the clinical and socioeconomic profile of children who die with community-acquired RSV infection globally. To gain insight into the clinical characteristics of RSV-related mortality in young children, we initiated the RSV Global Online Mortality Database (RSV GOLD) study with the aim to gather available retrospective data for fatal community-acquired RSV infections across the world.

## Methods

### Study design and patients

RSV GOLD is a global study that retrospectively analysed individual data for children aged 0–59 months who died with community-acquired RSV infection between Jan 1, 1995, and Oct 31, 2015. We identified research groups through a comprehensive literature search on PubMed (for articles published up to Feb 3, 2015) using the key terms “RSV”, “respiratory syncytial virus”, or “respiratory syncytial viral” combined with “mortality”, “fatality”, “death”, “died”, “deaths”, or “CFR”. We limited the search to papers written in English with the abstract and full text available. We invited authors of scientific, peer-reviewed papers reporting RSV-related mortality in children to collaborate. Additionally, we obtained unpublished individual patient data from researchers and clinicians who we identified via research networks. We invited researchers and clinicians to participate in this study between Nov 1, 2014, and Oct 31, 2015. We included data for children with laboratory-confirmed RSV infection and excluded cases of nosocomial or post-stem cell transplantation RSV infection. Since this study only used anonymised secondary data, the institutional research board of the University Medical Centre Utrecht waived the requirement for parental informed consent.

### Procedures

We collected data using an online questionnaire designed by a group of investigators (NMS, LJB, HN, FPP, BDG, and DEG). The questionnaire aimed to collect information about clinical and socioeconomic characteristics of children with RSV-related mortality. Characteristics of interest were based on risk factors for RSV-related and pneumonia-related mortality as described in the literature and on expert opinion (appendix).

We carefully validated patient information by direct communication with participating researchers for data entry errors, missing variables, and conflicting data. We deemed age at death within 0–59 months, laboratory-confirmed RSV diagnosis, and community-acquired RSV infection minimum essential data for inclusion. After assessment of data quality, we excluded children if nosocomial infection could not be ruled out on the basis of available clinical information or if death occurred before 1995. We sent additional questions about admission to paediatric intensive care units and availability of mechanical ventilation to the participating researcher if this information could not be extracted from the shared data.

We defined the following patient populations: children with comorbidities, healthy term children, and preterm children without other comorbidities (healthy preterm children). Children with comorbidities had at least one underlying disease, such as congenital heart disease, a genetic or chromosomal disorder, HIV infection, or active tuberculosis. Healthy term children were born without comorbidities at 37 weeks' gestational age or later and healthy preterm children were born without comorbidities at earlier than 37 weeks' gestational age. When data for comorbidities or prematurity were not recorded, we assumed that the children were healthy term. We categorised countries as high income, upper middle income, lower middle income, or low income on the basis of the World Bank classifications for 2016.^22^ We considered Hong Kong and Taiwan high-income countries in line with the World Bank classification. The composite variable neurological disease consisted of neurodevelopmental, neuromuscular, and other neurological disease, such as epilepsy or encephalopathy.

### Statistical analysis

We calculated descriptive statistics for all variables. We provide frequencies and proportions for categorical variables. We report the summary results for continuous variables as medians with IQRs. We analysed data by World Bank income regions. Additionally, we analysed data separately for children with comorbidities, healthy term children, and healthy preterm children. Using WHO child growth standards,^23^ we calculated *Z* scores for weight for age, height for age, and weight for height. We did not correct weight for age for gestational age.

To rule out bias, we did sensitivity analyses excluding cases with missing data for prematurity or comorbidities to test our assumption that children with missing data were healthy term. Similarly, we analysed to what extent our results were sensitive to the contribution of a large number of cases from Argentina and Kenya by doing analysis without these countries. We tested meaningful subgroup differences for significance with Kruskal-Wallis or χ^2^ statistical tests. We did further paired testing between subgroups using Mann-Whitney *U* or χ^2^ tests with Bonferroni correction for multiple testing. We did not test subgroup differences of results presented in the appendix for significance. We did all statistical analyses with SPSS statistical software (version 22.0).

### Role of the funding source

The funder had no role in study design, data collection, data analysis, data interpretation, or writing of the report. NMS and LJB had full access to all the data in the study and the corresponding author had final responsibility for the decision to submit for publication.

## Results

**Figure 1:**
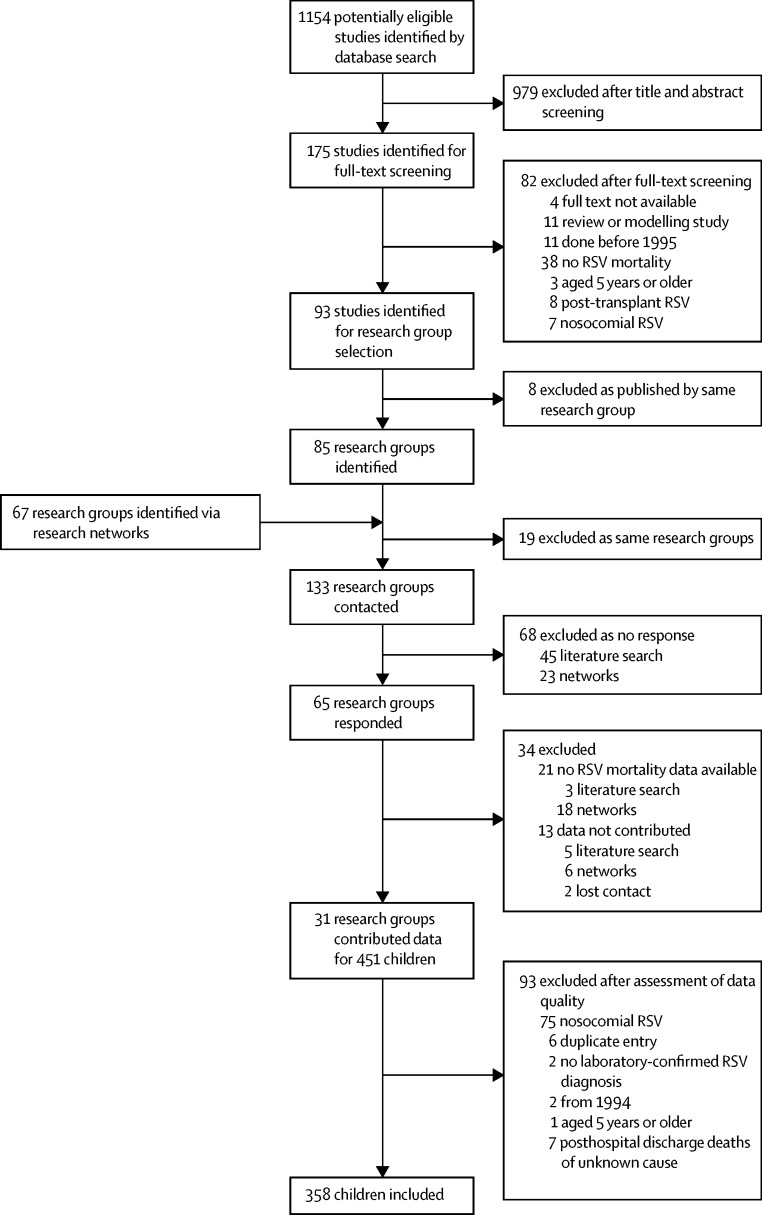
Study selection RSV=respiratory syncytial virus.

**Figure 2:**
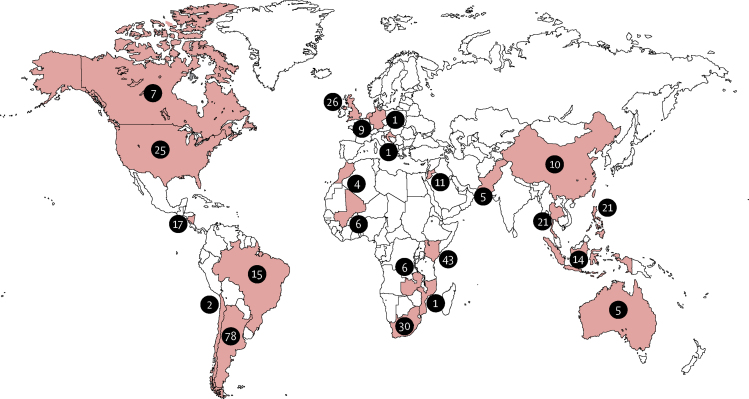
Locations of the respiratory syncytial virus-related deaths in young children included in the analysis Numbers of deaths are given for each country included.

The literature search resulted in 1154 publications (figure 1). From these publications, we identified 85 research groups, which we complemented with research groups identified through existing research networks, particularly through the RSV Global Epidemiology Network.^5^ We obtained data for 451 children with RSV-related mortality from the 31 research groups that shared data. We obtained both published and unpublished cases. Using original publications to contact research groups, we estimated that we could have missed a maximum of 140 cases from the published literature as most researchers who did not respond to our invitation to participate had reported solitary cases of RSV-related mortality: in 28 publications, the number of potential RSV-related deaths was mentioned and in 15 (54%) of those, one or two RSV-related deaths were described. Many non-responders were research groups from high-income countries (41 [60%] of 68) and had published their results between 2010 and 2015 (21 [47%] of 45). Publications used to identify research groups that contributed data are shown in the appendix. After initial assessment of the 451 children, we excluded 93 (21%). Most exclusions were associated with nosocomial RSV infection. We excluded six (1%) duplicate entries. We included 358 (79%) children with RSV-associated mortality originating from 23 different countries across the world (figure 2, appendix). RSV diagnosis was established most often by immunofluorescence or PCR (appendix). PCR was mostly used in children who died after 2005. Clear seasonality with an annual peak was reported in 18 (78%) of the 23 countries.

117 (33%) children were from low-income or lower middle-income countries, 77 (22%) were from upper middle-income countries, and 164 (46%) were from high-income countries (table). 190 (53%) children were male; we observed no sex differences between countries of different incomes. Children from high-income countries had a higher median age at death than did those in low-income or lower middle-income countries and children from high-income countries had stayed in hospital for a longer period and were more often admitted to a paediatric intensive care unit than those from upper middle-income countries or low-income or lower middle-income countries. Median time between onset of symptoms and death was longer in children from high-income countries (21·0 days [IQR 12·0–37·0]) than in those from low-income or lower middle-income countries (9·5 days [7·0–16·8]) or upper middle-income countries (10·0 days [8·0–14·0]; appendix).

**Table:** Clinical characteristics and risk factors in RSV-related child deaths

		**Low-income or lower middle-income countries (n=117)**	p value*	**Upper middle-income countries (n=77)**	**p value**†	**High-income countries (n=164)**	**p value**‡
Male sex		58 (50%)	0·976	38 (49%)	0·247	94 (57%)	0·199
Age at death (months)		5·0 (2·3–11·0)	0·973	4·0 (2·0–10·0)	0·023	7·0 (3·6–16·8)	0·006
Younger than 6 months at death		68 (58%)	0·893	44 (57%)	0·014	66 (40%)	0·003
Prematurity§		9 (8%)	0·083	12 (16%)	0·043	45 (27%)	0·000
Gestational age (weeks)		38·0 (38·0–39·0); n=22	0·144	38·0 (34·5–38·0); n=38	0·250	38·1 (32·1–40·0); n=80	0·688
Comorbidity§		33 (28%)	0·008	36 (47%)	0·001	114 (70%)	0·000
Oxygen saturation on room air at hospital admission (%)		92·0% (85·8–97·3); n=94	0·022	87·0% (85·0–94·0); n=47	0·448	89·0% (76·5–91·0); n=37	0·001
Weight for age *Z* score of less than −2		64/111 (58%)	0·924	33/58 (57%)	0·370	49/99 (50%)	0·236
Contact with health-care provider before admission to hospital		28/65 (43%)	0·870	17/38 (45%)	0·123	41/68 (60%)	0·047
Time between onset of symptoms and admission (days)		5·0 (3·0–7·0); n=71	0·002	3·0 (2·0–4·0); n=45	0·387	3·0 (1·0–5·0); n=135	0·000
Length of stay in hospital (days)		3·0 (2·0–6·0)	0·000	7·0 (3·0–11·5)	0·000	15·0 (7·0–32·0); n=149	0·000
Availability of intensive care unit		28 (24%)	0·000	75 (97%)	0·038	164 (100%)	0·000
Intensive care unit admission		23/116 (20%)	0·000	27/56 (48%)	0·000	152 (93%)	0·000
Mechanical ventilation		23/114 (20%)	0·000	33/60 (55%)	0·000	138/155 (89%)	0·000
Urban living area		22/66 (33%)	0·000	39/40 (98%)	0·867	129/133 (97%)	0·000
At least one sibling present in household		30/39 (77%)	0·122	16/17 (94%)	0·003	62/109 (57%)	0·027
Time of death relative to RSV seasonality							
	Death during RSV season	61/79 (77%)	0·004	35/64 (55%)	0·000	122/134 (91%)	0·005
	Death within 1 month before or after RSV season	7/79 (9%)	0·050	13/64 (20%)	0·001	7/134 (5%)	0·303

Data are n (%), median (IQR), or n/N (%). Statistical comparisons with Mann-Whitney *U* test or χ^2^ test with p values of less than 0·0167 taken to be significant according to Bonferroni correction for multiple testing. RSV=respiratory syncytial virus.

* Low-income or lower middle-income country versus upper middle-income country.

† Upper middle-income country versus high-income country.

‡ Low-income or lower middle-income country versus high-income country.

§ Considered absent when missing.

We analysed the prevalence of comorbidities in our study (table) and distinguished clinical characteristics of children with and without comorbidities (appendix). Data for comorbidities were missing for some children. Available data showed that comorbidities were present in at least 183 (51%) children. Congenital heart disease was the most frequent comorbid condition identified. The proportion of children with comorbidities was similar during sensitivity analyses when children with missing data (for comorbidities and gestational age) were excluded, except that the proportion with comorbidities in low-income or lower middle-income countries was more than doubled (appendix). Specific information about the presence of comorbidities and prematurity was available in 243 (68%) children. The proportion of missing information about comorbidities and prematurity was similar for children aged younger than 6 months at the time of death (54 [30%] of 178) and older children (61 [34%] of 180).

**Figure 3:**
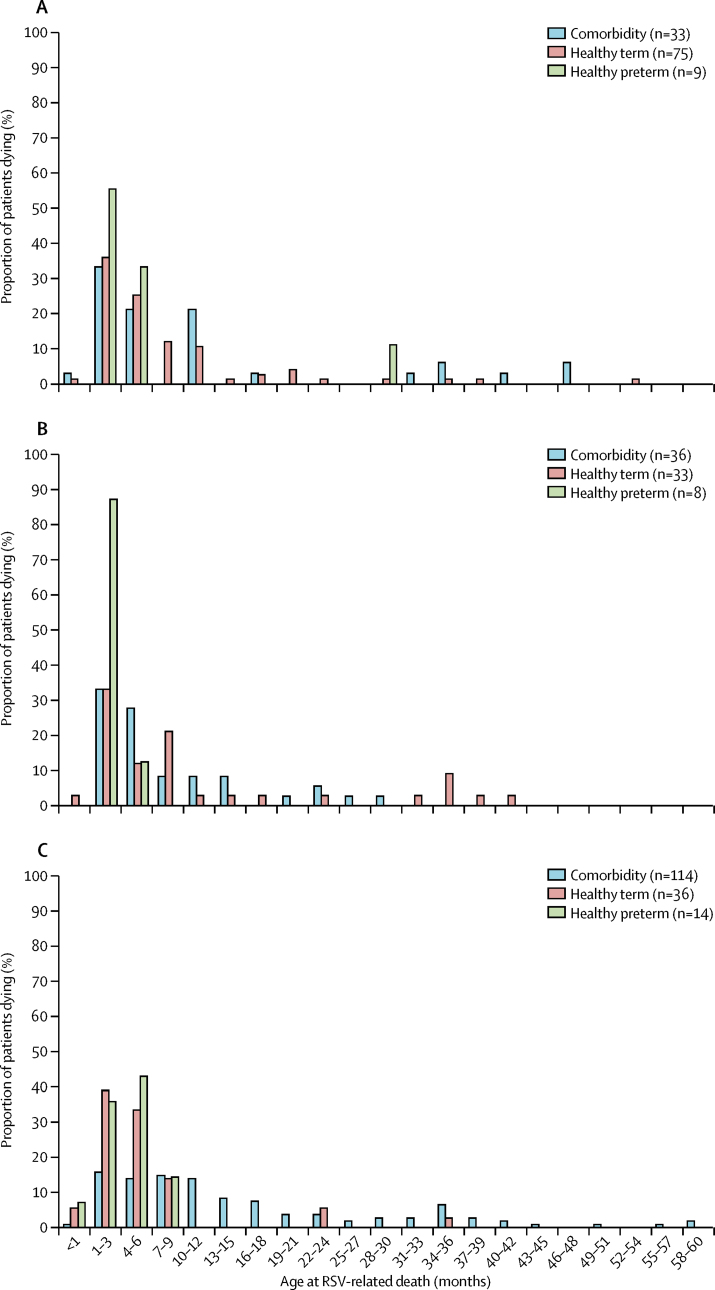
Age distribution at the time of RSV-related death in children Low-income or lower middle-income countries (A), upper middle-income countries (B), and high-income countries (C). RSV=respiratory syncytial virus.

We analysed how the presence of comorbidities affected age at death (figure 3). Most healthy term and healthy preterm children were aged younger than 6 months at the time of RSV-related death (appendix). The proportion of children aged younger than 6 months at the time of death was even larger when only children with complete data for comorbidity and gestational age status were included than when all children were included (appendix). Age at death of children with comorbidities from high-income countries was higher than that of those from low-income or middle-income countries.

We analysed the proportion of children with low weight for age. More than half of all children had a weight for age of less than −2 SDs (table). In high-income countries, this finding was mainly explained by a large proportion of preterm children and children with comorbidities. Only eight (28%) of 29 healthy term children in high-income countries had low weight for age compared with eight (89%) of nine healthy preterm children and 33 (54%) of 61 children with comorbidities (appendix). By contrast, in the other two income regions, we found a high proportion (≥48%) of low weight for age in all three children groups, including healthy term infants.

Data for co-infection were scarce. Microbiological data other than RSV diagnosis were sparsely available. We identified co-pathogens in 70 children, with 22 (31%) who tested positive for two or more pathogens besides RSV (data not shown). Positive blood cultures were reported in four (6%) children (two [3%] with *Pseudomonas aeruginosa*, one [1%] with *Klebsiella* spp, and one [1%] with *Staphylococcus aureus*).

Data from Argentina (78 [22%]) and Kenya (43 [12%]) contributed to about one third of all RSV-related deaths (121 [34%]). We did a sensitivity analysis excluding these data. The results from these analyses showed a higher age at death for children from high-income countries (10·0 months [4·0–24·0]) than from upper middle-income (4·0 months [2·0–10·0]) or low-income or lower middle-income (4·0 months [2·2–7·0]) countries, which was the same trend as for the main analysis. Other clinical characteristics, including sex and the proportion of prematurity or comorbidities, were also unchanged from the main analysis (data not shown).

## Discussion

Severe RSV infection is one of the major causes of global mortality in young children, with 99% of deaths occurring in developing countries in 2015.^5^ RSV GOLD is the first descriptive study of global RSV-related mortality using individual case records. We studied clinical characteristics of children who died with RSV in hospitals across the world. We supplemented data from previously published cases of RSV-related in-hospital death with unpublished data by contacting individual investigators, research groups, and networks. Complete clinical information was generally available for children from high-income countries, but clinical data from low-income and middle-income countries were often incomplete. For the first time, we showed that a substantial proportion of in-hospital RSV-related deaths in young children occurred in those with severe comorbidities. In low-income and middle-income countries, most children who died with RSV infection were aged younger than 6 months.

This study describes, to our knowledge, the largest number of children with RSV-related mortality to date. We obtained good global representation, with coverage of all six continents. Clinical profiles of children who die with RSV are essentially different in lower-income countries, where most childhood RSV-associated mortality occurs.^1^, ^5^ In low-income or lower middle-income countries, we found that at least 28% of deaths occurred in children with severe comorbidities, such as congenital heart disease. Our data suggest that most RSV-related in-hospital deaths in both low-income and middle-income countries occur in children aged younger than 6 months. Most children with comorbidities (eg, severe prematurity or congenital heart disease) in these countries might already have died during neonatal age. Additionally, a longer time interval between the onset of symptoms and admission to hospital in low-income countries than in middle-income and high-income countries and a lower proportion of children being admitted to intensive care units and receiving mechanical ventilation in low-income and middle-income countries than in high-income countries suggest that high RSV mortality in these countries might reflect limitations in health-care quality or access to care rather than specific susceptibility of the children. Young age at death of children with comorbidities in low-income and middle-income countries might have been related to low access to care as well. Paediatric intensive care was available to only 24% of children from low-income or lower middle-income countries and to most children from upper middle-income countries, although only 48% of these children were actually admitted to the intensive care unit.

The role of comorbidities in RSV-related childhood mortality has mostly been described in high-income countries.^10^, ^11^, ^24^ The differences in characteristics of children with or without comorbidities were in line with previously published data from high-income settings. Previous studies^10^, ^11^, ^13^, ^14^, ^25^, ^26^ have also described a higher age of children with comorbidities at admission or death than of previously healthy children, whereas young age is a risk factor for severe RSV disease in otherwise healthy preterm children.^27^ For high-income countries, we substantiate that the age distribution of children who die with RSV infection is substantially different between children with and without severe comorbidities.

Several strategies to prevent RSV infection and RSV-related mortality are being developed.^28^ Age at death could influence the effectiveness of perinatal immunisation strategies, including passive immunisation by maternal vaccination during pregnancy and administration of RSV-specific long-half-life monoclonal antibody at birth. Our age distribution data suggest that maternal vaccination will be more effective for healthy term children than for children with comorbidities because antibody concentrations decrease after birth over time. Our data suggest that children with comorbidities will need additional preventive interventions, such as extended passive immunisation or infant vaccination to optimise prevention of RSV-related mortality.

A key strength of this study is that we obtained most previously published cases of RSV-related mortality in children and supplemented this data with data sourced through existing networks, leading to a sample size large enough to draw robust conclusions and do some sensitivity analyses. Second, we verified the quality of each case record through direct interaction with the local researchers. Finally, RSV GOLD is a case series with global representation. Limitations also deserve discussion. First, although RSV GOLD forms, to our knowledge, the largest case series of RSV-related childhood mortality in hospital, this case series still reflects only a small proportion of all RSV-related deaths worldwide, as most global acute lower respiratory tract infection deaths occur outside of hospital.^29^ Data are scarce for RSV-related deaths in the community. Second, in RSV GOLD, only 117 (33%) of the children studied were from low-income or lower middle-income countries, which contrasts with the estimate that 99% of RSV-related mortality occurs in developing countries.^5^ Third, RSV GOLD is a case series, which does not allow for calculation of effect sizes of risk factors for RSV-associated mortality. A population-based study^16^ from Buenos Aires reported sepsis and pneumothorax as clinical risk factors for RSV-associated mortality, although sepsis was not pathogen specific. To calculate risk ratios for global RSV-related death with use of original case records, a large prospective population-based study would be needed, which would be challenging given the estimated RSV-attributable mortality rate of 0·86–0·94 per 1000 livebirths.^16^ Fourth, our data are limited by several biases. Data are primarily from research sites with availability of diagnostic tests for RSV and RSV diagnostics changing over time, with more sensitive molecular diagnostics used after 2005 than before 2005 (selection bias). Differences in the proportion of available data between income regions could have caused additional selection bias. For example, white blood count and haemoglobin concentration were more often available in low-income regions, which probably reflects measurements in a clinical research setting. Some authors who published data for RSV-related deaths did not respond (response bias), and data for prematurity and comorbidities were often missing, particularly from low-income and middle-income countries (misclassification bias). Misclassification bias could have led to inaccurate interpretation of results. The estimated prevalence of comorbidities in children from low-income or lower middle-income countries is probably an underestimation caused by missing data for comorbidities, but could also be an overestimation caused by selection bias due to a research setting, for example. However, results from our sensitivity analysis supported the main results of our study. Finally, since a substantial proportion of sociodemographic data were missing, these data should be interpreted with caution. For example, the high proportion of RSV-related deaths outside of the RSV season in some countries could reflect inaccurate seasonality data, but could also result from year-round RSV activity, as most of these countries had subtropical climates.

RSV-related infection is an important cause of death in children aged younger than 5 years. Data for RSV-related deaths are scarce and, when available, these data are limited to in-hospital deaths in research settings. Additionally, data for some of the key variables, such as comorbidities and gestational age, are missing. RSV GOLD is being further expanded to collect data for RSV-related deaths from any health facility or community-based study worldwide. Investments to understand the cause of death in young children need to be sustained to inform vaccine policy, particularly in low-income and middle-income settings.
