# Correction to *Lancet Glob Health* 2017; 5: e984–91

**DOI:** 10.1016/S2214-109X(17)30382-0

**Published:** 2017-10-03

*Scheltema NM, Gentile A, Lucion F, et al. Global respiratory syncytial virus-associated mortality in young children (RSV GOLD): a retrospective case series.* Lancet Glob Health *2017; **5:** e984–91. http://dx.doi.org/10.1016/S2214-109X(17)30344-3*—The following sentence in the Findings section of the Summary should have read: Median age for RSV-related deaths was 5·0 months (IQR 2·3–11·0) in low-income or lower middle-income countries, 4·0 months (2·0–10·0) in upper middle-income countries, and 7·0 months (3·6–16·8) in high-income countries.” This correction has been made to the online version as of Oct 3, 2017.

