# Supplementary material for: Global respiratory syncytial virus-associated mortality in young children (RSV GOLD): a retrospective case series
Source: Lancet Glob Health. 2017 Sep 11;5(10):e984–91. doi: 10.1016/S2214-109X(17)30344-3 (PMC5599304; doi:10.1016/S2214-109X(17)30344-3)
Supplement: Supplementary appendix [file mmc1.pdf]

THE LANCET
Global Health

Supplementary appendix

This appendix formed part of the original submission and has been peer reviewed. We post it as supplied by the authors.

Supplement to: Scheltema NM, Gentile A, Lucion F, et al. Global respiratory syncytial virus-associated mortality in young children (RSV GOLD): a retrospective case series. *Lancet Glob Health* 2017; **5:** e984–91.

Supplemental Figure 1. Sensitivity analysis for age distribution at RSV-related death excluding children with missing data for comorbidity or prematurity

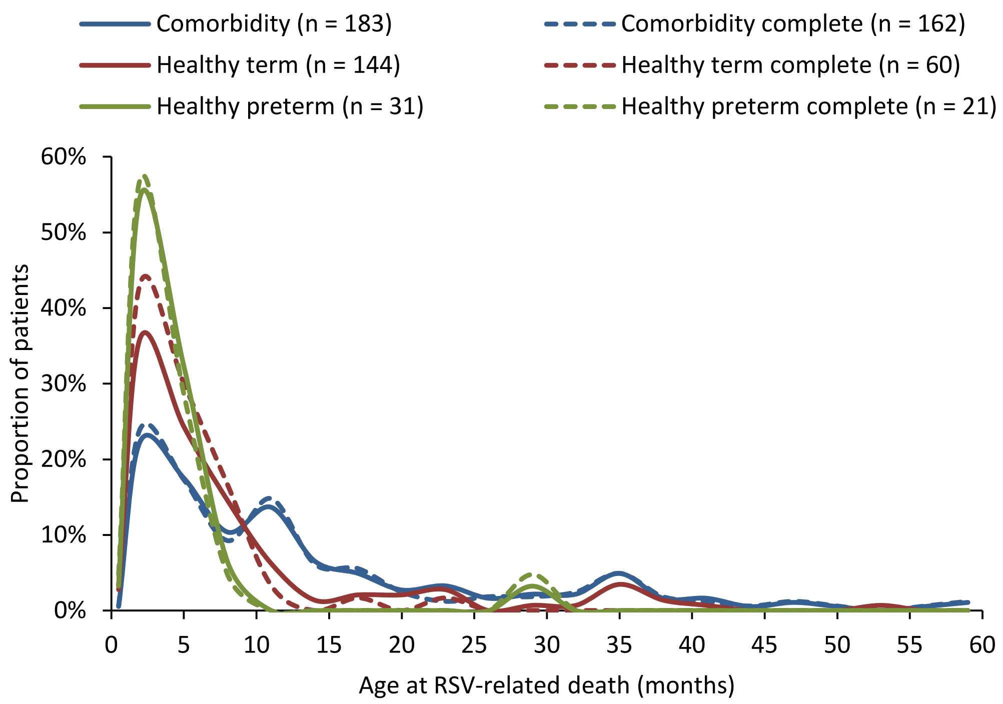

Supplemental Table 1. Publications that identified research groups that contributed data

Title	Authors	Journal	Year published
Viral etiology of severe pneumonia among Kenyan infants and children	Berkley et al.	JAMA	2010
The impact of prophylaxis on paediatric intensive care unit admissions for RSV infection: a retrospective, single-centre study	Butt et al.	Eur J Pediatr	2011
Intravenous palivizumab and ribavirin combination for respiratory syncytial virus disease in high-risk pediatric patients	Chávez-Bueno et al.	Pediatr Infect Dis J	2007
Epidemiological study of hospitalization associated with respiratory syncytial virus infection in Taiwanese children between 2004 and 2007	Chi et al.	J Formos Med Assoc	2011
Epidemiology of viral-associated acute lower respiratory tract infection among children <5 years of age in a high HIV prevalence setting, South Africa, 2009-2012	Cohen et al.	Pediatr Infect Dis J	2014
Viral epidemiology of respiratory infections among children at a tertiary hospital in Southern Brazil	de-Paris et al.	Rev Soc Bras Med Trop	2014
Incidence and clinical features of hospitalization because of respiratory syncytial virus lower respiratory illness among children less than two years of age in a rural Asian setting	Djelantik et al.	Pediatr Infect Dis J	2003
Case fatality proportions and predictive factors for mortality among children hospitalized with severe pneumonia in a rural developing country setting	Djelantik et al.	J Trop Pediatr	2003
Transaminase levels in ventilated children with respiratory syncytial virus bronchiolitis	Eisenhut et al.	Intensive Care Med	2004
Macronutrients during pregnancy and life-threatening respiratory syncytial virus infections in children.	Ferolla et al.	Am J Respir Crit Care Med	2013
Single, dual and multiple respiratory virus infections and risk of hospitalization and mortality	Goka et al.	Epidemiol Infect	2015
Premorbid factors and outcome associated with respiratory virus infections in a pediatric intensive care unit	Hon et al.	Pediatr Pulmonol	2008
Survey of severe respiratory syncytial virus infection in Kyoto Prefecture from 2003 to 2007	Ito et al.	Pediatr Int	2010
The epidemiology and aetiology of infections in children admitted with clinical severe pneumonia to a university hospital in Rabat, Morocco	Jroundi et al.	J Trop Pediatr	2014
Pre-existing disease is associated with a significantly higher risk of death in severe respiratory syncytial virus infection	K. Thorburn	Arch Dis Child	2009
Burden of respiratory syncytial virus in hospitalized infants and young children in Amman, Jordan	Khuri-Bulos et al.	Scand J Infect Dis	2010
Risk factors associated with death in patients with severe respiratory syncytial virus infection	Lee et al.	J Microbiol Immunol Infect	2014
Epidemiology and risk factors for severe respiratory syncytial virus infections requiring pediatric intensive care admission in Hong Kong children	Leung et al.	Infection	2013
Respiratory syncytial virus: clinical and epidemiological pattern in pediatric patients admitted to a children's hospital between 2000 and 2013	Lucion et al.	Arch Argent Pediatr	2014
Hospital-acquired viral infection increases mortality in children with severe viral respiratory infection	M.C. Spaeder, J.C. Fackler	Pediatr Crit Care Med	2011
Five-year cohort study of hospitalization for respiratory syncytial virus associated lower respiratory tract infection in African children	Madhi et al.	J Clin Virol	2006
Differing manifestations of respiratory syncytial virus-associated severe lower respiratory tract infections in human immunodeficiency virus type 1-infected and uninfected children	Madhi et al.	Pediatr Infect Dis J	2000
Epidemiology of respiratory syncytial virus-associated acute lower respiratory tract infection hospitalizations among HIV-infected and HIV-uninfected South African children, 2010–2011	Moyes et al.	J Infect Dis	2013
Hospitalisation for acute lower respiratory tract infection due to respiratory syncytial virus in Thailand, 2008-2011	Naorat et al.	J Infect Dis	2013
Etiology and epidemiology of viral pneumonia among hospitalized children in rural Mozambique: a malaria endemic area with high prevalence of human immunodeficiency virus	O'Callaghan-Gordo et al.	Pediatr Infect Dis J	2011
Bronchiolitis caused by respiratory syncytial virus in the period from 2003 to 2009	Roglić et al.	Croatian J of infection	2009
Respiratory viruses from hospitalized children with severe pneumonia in the Philippines	Suzuki et al.	BMC Infect Dis	2012

Supplemental Table 2. Origin of data for children younger than five years with RSV-related death

Country	Children with comorbidity (n = 183)	Healthy term children (n = 144)	Healthy preterm children (n = 31)
Low-income			
Mali	3 (2%)	1 (1%)	2 (6%)
Mozambique	0	1 (1%)	0
Lower middle-income			
Indonesia	0	14 (10%)	0
Kenya	17 (9%)	23 (16%)	3 (10%)
Morocco	0	4 (3%)	0
Nicaragua	7 (4%)	8 (6%)	2 (6%)
Pakistan	3 (2%)	1 (1%)	1 (3%)
Philippines	1 (1%)	20 (14%)	0
Zambia	2 (1%)	3 (2%)	1 (3%)
Upper middle-income			
Brazil	8 (4%)	5 (3%)	2 (6%)
Jordan	4 (2%)	5 (3%)	2 (6%)
South Africa	18 (10%)	8 (6%)	4 (13%)
Thailand	6 (3%)	15 (10%)	0
High-income			
Argentina	43 (23%)	26 (18%)	9 (29%)
Australia	5 (3%)	0	0
Canada	4 (2%)	3 (2%)	0
Chile	2 (1%)	0	0
China (Hong Kong & Taiwan)	10 (5%)	0	0
Croatia	1 (1%)	0	0
Germany	1 (1%)	0	0
The Netherlands	7 (4%)	1 (1%)	1 (3%)
United Kingdom	23 (13%)	2 (1%)	1 (3%)
USA	18 (10%)	4 (3%)	3 (10%)

Data are n (%). RSV=respiratory syncytial virus.

Supplemental Table 3. Frequency of RSV diagnostics used over study duration per income region

**RSV diagnostic test used**	**1995 - 2000 (n = 39)**	**2001 - 2005 (n = 117)**	**2006 - 2010 (n = 140)**	**2011 - 2015 (n = 112)**
Low-income or lower middle-income country (n = 122)				
PCR	0	2	16	27
Immunofluorescence	0	11	20	27
Enzyme Immuno assay	6	8	0	1
Culture	0	2	0	2
Serology	0	0	0	0
Other	0	0	0	0
Upper middle-income country (n = 122)				
PCR	0	3	30	16
Immunofluorescence	14	6	3	5
Enzyme Immuno assay	0	0	0	0
Culture	0	3	17	1
Serology	0	3	17	1
Other	0	0	0	3
High-income country (n = 164)				
PCR	0	0	6	8
Immunofluorescence	13	62	23	20
Enzyme Immuno assay	2	15	6	0
Culture	4	1	1	0
Serology	0	0	0	0
Other	0	1	1	1

Data are number. RSV=respiratory syncytial virus.

Supplemental Table 4. Additional characteristics and risk factors in RSV-related child deaths

	Low-income or lower middle-income countries (n = 117)	Upper middle-income countries (n = 77)	High-income countries (n = 164)
Comorbidity	33 (28%)	36 (47%)	114 (70%)
Genetic or chromosomal disease	4 (3%)	3 (4%)	31 (19%)
Down's syndrome	3 (3%)	2 (3%)	10 (6%)
Congenital heart disease	15 (13%)	9 (12%)	43 (26%)
Neurological disease	4 (3%)	5 (6%)	40 (24%)
Chronic lung disease	1 (1%)	0	45 (27%)
Airway abnormality	0	1 (1%)	7 (4%)
Primary immunodeficiency	0	1 (1%)	7 (4%)
Malignancy	0	0	2 (1%)
HIV infection	5 (4%)	18 (23%)	0
Tuberculosis infection	1 (1%)	2 (3%)	0
Other underlying disease	9 (8%)	4 (5%)	4 (2%)
Biliary atresia	0	1 (1%)	0
Liver disease	0	1 (1%)	0
Renal disease	0	1 (1%)	2 (1%)
Immunosuppression	5 (4%)	0	0
Congenital abnormality	3 (3%)	0	0
Adenoid cyst	1 (1%)	0	0
Metabolic disorder	0	1 (1%)	2 (1%)
Clinical symptoms			
Cough	105 (90%)	67 (87%)	43 (26%)
Difficult breathing	105 (90%)	48 (62%)	68 (41%)
Fast breathing*	72 (62%)	41 (53%)	46 (28%)
Chest indrawing	83 (71%)	28 (36%)	36 (22%)
Central cyanosis	15 (13%)	8 (10%)	13 (8%)
Severe respiratory distress	27 (23%)	4 (5%)	46 (28%)
Inability to drink	25 (21%)	16 (21%)	31 (19%)
Lethargy or unconsciousness	38 (32%)	11 (14%)	14 (9%)
RSV diagnostic test used			
PCR	45 (38%)	49 (64%)	14 (9%)
Immunofluorescence	58 (50%)	28 (36%)	118 (72%)
Enzyme immunoassay	15 (13%)	0	23 (14%)
Culture	4 (3%)	21 (27%)	6 (4%)
Serology	0	21 (27%)	0
Other	0	3 (4%)	3 (2%)
RSV diagnostic specimen used			
Nasal swab	0	0	3 (2%)
Nasopharyngeal swab	60 (51%)	36 (47%)	13 (8%)
Nasopharyngeal aspirate	22 (19%)	35 (45%)	119 (73%)
Wash	35 (30%)	3 (4%)	0
Blood	0	21 (27%)	0
Other	2 (2%)	0	7 (4%)
White blood cell count			
< 6000 cu/mm	14 (12%)	6 (8%)	11 (7%)
6000-25000 cu/mm	81 (69%)	32 (42%)	49 (30%)
> 25000 cu/mm	13 (11%)	1 (1%)	9 (5%)
Unknown	6 (5%)	38 (49%)	95 (58%)
Haemoglobin level			
< 5 g/dL	3 (3%)	0	1 (1%)
5-8 g/dL	16 (14%)	5 (6%)	3 (2%)
8-10 g/dL	47 (40%)	7 (9%)	30 (18%)
> 10 g/dL	41 (35%)	32 (42%)	28 (17%)
Unknown	7 (6%)	33 (43%)	102 (62%)
Length of stay at intensive care unit (days)	3·5 (1·8-8·5, n = 38)	11·0 (6·0-22·0, n = 15)	13·0 (6·0-31·5, n = 132)
Duration of mechanical ventilation (days)	4·0 (1·0-9·3, n = 22)	8·0 (2·0-17·0, n = 19)	12·0 (5·0-27·3, n = 126)
Time between onset of symptoms and death (days)	9·5 (7·0-16·8, n = 72)	10·0 (8·0-14·0, n = 45)	21·0 (12·0-37·0, n = 135)
Type of feeding during first four months of life			
Exclusive breast feeding	23 (20%)	5 (6%)	11 (7%)
Mixed breast bottle feeding	16 (14%)	6 (8%)	18 (11%)
Exclusive bottle feeding	2 (2%)	6 (8%)	25 (15%)
Unknown	76 (65%)	60 (78%)	110 (67%)
Parental smoking	5/39 (13%)	5/15 (33%)	16/45 (36%)
Maternal education (highest level attended)			
No education	8 (7%)	4 (5%)	0
Primary school	8 (7%)	8 (10%)	4 (2%)

Secondary school	17 (15%)	9 (12%)	32 (20%)
University level	3 (3%)	1 (1%)	3 (2%)
Unknown	81 (69%)	55 (71%)	125 (76%)
Daycare attendance	3/38 (8%)	0/17	5/117 (4%)

Data are n (%), median (IQR), or n/N (%). RSV=respiratory syncytial virus. *For age younger than 2 months, at least 60 breaths per min; for age 2–11 months, at least 50 breaths per min; for age 1–5 years, at least 40 breaths per min.

Supplemental Table 5A. Clinical characteristics and risk factors in RSV-related child deaths from low-income or lower middle-income countries

	Children with comorbidity (n = 33)	Healthy term children (n = 75)	Healthy preterm children (n = 9)
Male sex	15 (45%)	38 (51%)	5 (56%)
Age at death (months)	5·0 (2·5-11·5)	5·0 (2·4-10·0)	3·0 (1·0-5·5)
Younger than 6 months at death	17 (52%)	44 (59%)	7 (78%)
Prematurity	0	NA	9 (100%)
Gestational age (weeks)	38·5 (38·0-39·8, n = 8)	38·0 (38·0-38·0, n = 11)	31·0 (31·0-31·5, n = 3)
Comorbidity			
Genetic or chromosomal disease	4 (12%)		
Congenital heart disease	15 (45%)		
Neurological disease	4 (12%)		
Chronic lung disease	1 (3%)		
Airway abnormality	0		
Primary immunodeficiency	0		
Malignancy	0		
HIV infection	5 (15%)		
Tuberculosis infection	1 (3%)		
Other underlying disease	9 (27%)		
Clinical symptoms			
Cough	30 (91%)	68 (91%)	7 (78%)
Difficult breathing	29 (88%)	68 (91%)	8 (89%)
Fast breathing	27 (82%)	39 (52%)	6 (67%)
Chest indrawing	27 (82%)	49 (65%)	7 (78%)
Central cyanosis	4 (12%)	9 (12%)	2 (22%)
Severe respiratory distress	9 (27%)	14 (19%)	4 (44%)
Inability to drink	9 (27%)	12 (16%)	4 (44%)
Lethargy or unconsciousness	9 (27%)	28 (37%)	1 (11%)
Oxygen saturation on room air at hospital admission (%)	92·0 (82·0-98·0, n = 31)	93·0 (87·0-97·0, n = 54)	92·0 (86·0-97·0, n = 9)
Weight for age Z-score of less than -2	23/31 (74%)	35/73 (48%)	6/7 (86%)
RSV diagnostic test used			
PCR	9 (27%)	32 (43%)	4 (44%)
Immunofluorescence	24 (73%)	29 (39%)	5 (56%)
Enzyme immunoassay	1 (3%)	14 (19%)	0
Culture	2 (6%)	2 (3%)	0
Serology	0	0	0
Other	0	0	0
RSV diagnostic specimen used			
Nasal swab	0	0	0
Nasopharyngeal swab	19 (58%)	37 (49%)	4 (44%)
Nasopharyngeal aspirate	7 (21%)	14 (19%)	1 (11%)
Wash	7 (21%)	26 (35%)	2 (22%)
Blood	0	0	0
Other	1 (3%)	0	1 (11%)
Contact with health-care provider before admission to hospital	12/25 (48%)	14/35 (40%)	2/5 (40%)
Time between onset of symptoms and admission (days)	3·0 (2·0-7·8, n = 26)	5·5 (3·0-7·0, n = 38)	4·0 (3·0-5·0, n = 7)
Length of stay in hospital (days)	4·0 (1·5-8·0)	3·0 (2·0-6·0)	3·0 (1·5-8·0)
Availability of intensive care unit	11 (33%)	14 (19%)	3 (33%)
Intensive care unit admission	9 (27%)	11/74 (15%)	3 (33%)
Mechanical ventilation	9 (27%)	11/72 (15%)	3 (33%)
Urban living area	9/21 (43%)	10/41 (24%)	3/4 (75%)
At least one sibling present	11/13 (85%)	15/20 (75%)	4/6 (67%)
Time of death relative to RSV seasonality			
Death during RSV season	21/25 (84%)	34/48 (71%)	6/6 (100%)
Death within 1 month before or after RSV season	1/25 (4%)	6/48 (13%)	0

Data are n (%), median (IQR), or n/N (%). RSV=respiratory syncytial virus. NA=not applicable.

Supplemental Table 5B. Clinical characteristics and risk factors in RSV-related child deaths from upper middle-income countries

	Children with comorbidity (n = 36)	Healthy term children (n = 33)	Healthy preterm children (n = 8)
Male sex	17 (47%)	17 (52%)	4 (50%)
Age at death (months)	4·2 (2·3-10·8)	7·0 (2·3-16·5)	2·0 (2·0-2·8)
Younger than 6 months at death	21 (58%)	15 (45%)	8 (100%)
Prematurity	4/27 (15%)	NA	8 (100%)
Gestational age (weeks)	38·0 (37·0-38·0, n = 21)	39·0 (37·5-40·0, n = 10)	31·0 (29·0-35·0, n = 7)
Comorbidity			
Genetic or chromosomal disease	3 (8%)		
Congenital heart disease	9 (25%)		
Neurological disease	5 (14%)		
Chronic lung disease	0		
Airway abnormality	1 (3%)		
Primary immunodeficiency	1 (3%)		
Malignancy	0		
HIV infection	18 (50%)		
Tuberculosis infection	2 (6%)		
Other underlying disease	4 (11%)		
Clinical symptoms			
Cough	30 (83%)	31 (94%)	6 (75%)
Difficult breathing	17 (47%)	25 (76%)	6 (75%)
Fast breathing	22 (61%)	16 (48%)	3 (38%)
Chest indrawing	16 (44%)	7 (21%)	5 (63%)
Central cyanosis	3 (8%)	4 (12%)	1 (13%)
Severe respiratory distress	2 (6%)	1 (3%)	1 (13%)
Inability to drink	10 (28%)	5 (15%)	1 (13%)
Lethargy or unconsciousness	6 (17%)	4 (12%)	1 (13%)
Oxygen saturation on room air at hospital admission (%)	87·0 (84·5-90·3, n = 25)	92·0 (86·5-96·5, n = 17)	87·0 (78·5-91·5, n = 5)
Weight for age Z-score of less than -2	18/29 (62%)	12/25 (48%)	3/4 (75%)
RSV diagnostic test used			
PCR	18 (50%)	27 (82%)	4 (50%)
Immunofluorescence	18 (50%)	6 (18%)	4 (50%)
Enzyme immunoassay	0	0	0
Culture	6 (17%)	15 (45%)	0
Serology	6 (17%)	15 (45%)	0
Other	1 (3%)	2 (6%)	0
RSV diagnostic specimen used			
Nasal swab	0	0	0
Nasopharyngeal swab	13 (36%)	21 (64%)	2 (25%)
Nasopharyngeal aspirate	20 (56%)	10 (30%)	5 (63%)
Wash	1 (3%)	2 (6%)	0
Blood	6 (17%)	15 (45%)	0
Other	0	0	0
Contact with health-care provider before admission to hospital	9/18 (50%)	6/16 (38%)	2/4 (50%)
Time between onset of symptoms and admission (days)	3·0 (2·0-4·8, n = 24)	4·0 (1·0-6·0, n = 15)	2·5 (1·8-4·0, n = 6)
Length of stay in hospital (days)	7·0 (3·3-11·8)	6·0 (3·0-10·0)	9·0 (5·3-15·3)
Availability of intensive care unit	36 (100%)	31 (94%)	8 (100%)
Intensive care unit admission	11/28 (39%)	10/20 (50%)	6/8 (75%)
Mechanical ventilation	16/22 (73%)	15/32 (47%)	2/6 (33%)
Urban living area	23/23 (100%)	10/11 (91%)	6/6 (100%)
At least one sibling present	8/8 (100%)	6/6 (100%)	2/3(67%)
Time of death relative to RSV seasonality			
Death during RSV season	16/31 (52%)	16/27 (59%)	3/6 (50%)
Death within 1 month before or after RSV season	9/31 (29%)	2/27 (7%)	2/6 (33%)

Data are n (%), median (IQR), or n/N (%). RSV=respiratory syncytial virus. NA=not applicable.

Supplemental Table 5C. Clinical characteristics and risk factors in RSV-related child deaths from high-income countries

	**Children with comorbidity (n = 114)**	**Healthy term children (n = 36)**	**Healthy preterm children (n = 14)**
Male sex	64 (56%)	21 (58%)	9 (64%)
Age at death (months)	11·0 (5·0-23·3)	4·0 (2·0-6·0)	3·8 (2·0-5·3)
Younger than 6 months at death	29 (25%)	26 (72%)	11 (79%)
Prematurity	31/105 (30%)	NA	14 (100%)
Gestational age (weeks)	38·1 (32·2-40·0, n = 62)	40·0 (39·0-40·0, n = 11)	30·0 (28·0-34·0, n = 7)
Comorbidity			
Genetic or chromosomal disease	31 (27%)		
Congenital heart disease	43 (38%)		
Neurological disease	40 (35%)		
Chronic lung disease	45 (39%)		
Airway abnormality	7 (6%)		
Primary immunodeficiency	7 (6%)		
Malignancy	2 (2%)		
HIV infection	0		
Tuberculosis infection	0		
Other underlying disease	4 (4%)		
Clinical symptoms			
Cough	34 (30%)	6 (17%)	3 (21%)
Difficult breathing	54 (47%)	9 (25%)	5 (36%)
Fast breathing	39 (34%)	6 (17%)	1 (7%)
Chest indrawing	28 (25%)	6 (17%)	2 (14%)
Central cyanosis	11 (10%)	1 (3%)	1 (7%)
Severe respiratory distress	37 (32%)	6 (17%)	3 (21%)
Inability to drink	2 (2%)	5 (14%)	1 (7%)
Lethargy or unconsciousness	12 (11%)	2 (6%)	0
Oxygen saturation on room air at hospital admission (%)	88·0 (75·8-92·8, n = 28)	89·0 (75·0-90·0, n = 7)	NA
Weight for age Z-score of less than -2	33/61 (54%)	8/29 (28%)	8/9 (89%)
RSV diagnostic test used			
PCR	13 (11%)	1 (3%)	0
Immunofluorescence	75 (66%)	31 (86%)	12 (86%)
Enzyme immunoassay	19 (17%)	3 (8%)	1 (7%)
Culture	3 (3%)	0	3 (21%)
Serology	0	0	0
Other	2 (2%)	1 (3%)	0
RSV diagnostic specimen used			
Nasal swab	1 (1%)	2 (6%)	0
Nasopharyngeal swab	11 (10%)	2 (6%)	0
Nasopharyngeal aspirate	81 (71%)	28 (78%)	10 (71%)
Wash	0	0	0
Blood	0	0	0
Other	6 (5%)	1 (3%)	0
Contact with health-care provider before admission to hospital	33/55 (60%)	7/10 (70%)	1/3 (33%)
Time between onset of symptoms and admission (days)	2·0 (1·0-3·0, n = 92)	5·0 (3·0-10·0, n = 31)	2·0 (2·0-3·0, n = 12)
Length of stay in hospital (days)	15·0 (6·0-34·6)	19·0 (9·0-32·0)	11·0 (6·5-23·5)
Availability of intensive care unit	114 (100%)	36 (100%)	14 (100%)
Intensive care unit admission	105 (92%)	34 (94%)	13 (93%)
Mechanical ventilation	94/107 (88%)	32/35 (91%)	12/13 (92%)
Urban living area	88/91 (97%)	31/32 (97%)	10/10 (100%)
At least one sibling present	40/70 (57%)	17/30 (57%)	5/9 (56%)
Time of death relative to RSV seasonality			
Death during RSV season	81/91 (89%)	31/32 (97%)	10/11 (91%)
Death within 1 month before or after RSV season	5/91 (5%)	1/32 (3%)	1/11 (9%)

Data are n (%), median (IQR), or n/N (%). RSV=respiratory syncytial virus. NA=not applicable.

Supplemental Table 6. Clinical characteristics and risk factors in RSV-related child deaths excluding children with missing data for comorbidity or prematurity

	**Low-income or lower middle-income countries (n = 45)**	**Upper middle-income countries (n = 48)**	**High-income countries (n = 150)**
Male sex	21 (47%)	23 (48%)	85 (57%)
Age at death (months)	4·0 (2·0-11·0)	3·0 (2·0-7·4)	7·0 (3·3-16·0)
Children with comorbidity	4·5 (2·0-11·3, n = 30)	4·0 (2·0-8·8, n = 27)	11·0 (5·0-22·0, n = 105)
Healthy term children	4·5 (1·5-8·3, n = 12)	2·8 (1·5-8·0, n = 14)	4·0 (1·9-5·3, n = 34)
Healthy preterm children	3 (2-15·5, n = 3)	2 (2-2, n = 7)	4·0 (2·0-5·0, n = 11)
Younger than 6 months at death	26 (58%)	35 (73%)	63 (42%)
Prematurity	3 (7%)	11 (23%)	42 (28%)
Gestational age (weeks)	38·0 (38·0-39·0, n = 21)	38·0 (35·0-38·0, n = 35)	39·0 (34·1-40·0, n = 77)
Comorbidity	30 (67%)	27 (56%)	105 (70%)
Genetic or chromosomal disease	3 (7%)	3 (6%)	28 (19%)
Congenital heart disease	12 (27%)	4 (8%)	41 (27%)
Neurological disease	3 (7%)	4 (8%)	38 (25%)
Chronic lung disease	1 (2%)	0	41 (27%)
Airway abnormality	0	1 (2%)	7 (5%)
Primary immunodeficiency	0	0	7 (5%)
Malignancy	0	0	2 (1%)
HIV infection	5 (11%)	16 (33%)	0
Tuberculosis infection	1 (2%)	2 (4%)	0
Other underlying disease	9 (20%)	3 (6%)	3 (2%)
Clinical symptoms			
Cough	41 (91%)	41 (85%)	40 (27%)
Difficult breathing	40 (89%)	25 (52%)	65 (43%)
Fast breathing	33 (73%)	22 (46%)	46 (31%)
Chest indrawing	34 (76%)	25 (52%)	36 (24%)
Central cyanosis	8 (18%)	7 (15%)	13 (9%)
Severe respiratory distress	16 (36%)	4 (8%)	44 (29%)
Inability to drink	13 (29%)	15 (31%)	31 (21%)
Lethargy or unconsciousness	11 (24%)	8 (17%)	13 (9%)
Oxygen saturation on room air at hospital admission (%)	92·0 (82·0-98·0, n = 41)	87·0 (85·5-89·3, n = 29)	89·0 (76·5-91·0, n = 37)
Weight for age Z-score of less than -2	31/43 (72%)	20/36 (56%)	48/97 (49%)
RSV diagnostic test used			
PCR	14 (31%)	27 (56%)	14 (9%)
Immunofluorescence	31 (69%)	21 (44%)	111 (74%)
Enzyme immunoassay	1 (2%)	0	23 (15%)
Culture	1 (2%)	0	1 (1%)
Serology	0	0	0
Other	0	2 (4%)	3 (2%)
RSV diagnostic specimen used			
Nasal swab	0	0	3 (2%)
Nasopharyngeal swab	20 (44%)	14 (29%)	13 (9%)
Nasopharyngeal aspirate	12 (27%)	30 (63%)	117 (78%)
Wash	7 (16%)	2 (4%)	0
Blood	0	21 (44%)	0
Other	1 (2%)	0	7 (5%)
Contact with health-care provider before admission to hospital	15/36 (42%)	8/18 (44%)	41/68 (60%)
Time between onset of symptoms and admission (days)	4·0 (3·0-7·0, n = 39)	3·0 (2·0-4·0, n = 43)	3·0 (1·0-5·0, n = 131)
Length of stay in hospital (days)	4·0 (1·5-10·0)	7·0 (4·0-11·0)	17·0 (7·8-32·3, n = 142)
Availability of intensive care unit	24 (53%)	48 (100%)	150 (100%)
Intensive care unit admission	17 (38%)	19/46 (41%)	138 (92%)
Mechanical ventilation	17/43 (40%)	12/31 (39%)	129/143 (90%)
Urban living area	19/32 (59%)	32/33 (97%)	127/131 (97%)
At least one sibling present in household	19/26 (73%)	13/14 (93%)	62/109 (57%)
Time of death relative to RSV seasonality			
Death during RSV season	28/37 (76%)	25/36 (69%)	119/131 (91%)
Death within 1 month before or after RSV season	2/37 (5%)	7/36 (19%)	7/131 (5%)

Data are n (%), median (IQR), or n/N (%). RSV=respiratory syncytial virus.

Supplemental Table 7. Frequency of age at RSV-related death per income region by comorbidity status

**Age at death**	**Children with comorbidity (n = 183)**	**Healthy term children (n = 144)**	**Healthy preterm children (n = 31)**
Low-income or lower middle-income country (n = 117)			
0-1 months of age	1 (3%)	1 (1%)	0
1-2 months of age	2 (6%)	9 (12%)	3 (33%)
2-3 months of age	5 (15%)	9 (12%)	1 (11%)
3-4 months of age	4 (12%)	9 (12%)	1 (11%)
4-5 months of age	4 (12%)	6 (8%)	0
5-6 months of age	1 (3%)	10 (13%)	2 (22%)
6-12 months of age	8 (24%)	18 (24%)	1 (11%)
12-24 months of age	2 (6%)	8 (11%)	0
24-60 months of age	6 (18%)	5 (7%)	1 (11%)
Upper middle-income country (n = 77)			
0-1 months of age	0	1 (3%)	0
1-2 months of age	1 (3%)	3 (9%)	0
2-3 months of age	8 (22%)	4 (12%)	6 (75%)
3-4 months of age	3 (8%)	4 (12%)	1 (13%)
4-5 months of age	7 (19%)	2 (6%)	0
5-6 months of age	2 (6%)	1 (3%)	1 (13%)
6-12 months of age	7 (19%)	9 (27%)	0
12-24 months of age	6 (17%)	3 (9%)	0
24-60 months of age	2 (6%)	6 (18%)	0
High-income country (n = 164)			
0-1 months of age	1 (1%)	2 (6%)	1 (7%)
1-2 months of age	2 (2%)	5 (14%)	0
2-3 months of age	11 (10%)	8 (22%)	4 (29%)
3-4 months of age	4 (4%)	1 (3%)	1 (7%)
4-5 months of age	5 (4%)	9 (25%)	4 (29%)
5-6 months of age	6 (5%)	1 (3%)	1 (7%)
6-12 months of age	31 (27%)	7 (19%)	3 (21%)
12-24 months of age	26 (23%)	0	0
24-60 months of age	28 (25%)	3 (8%)	0

Data are n (%). RSV=respiratory syncytial virus.

Supplemental RSV GOLD questionnaire

<u>RSV diagnosis.</u>
1.1 Diagnostic test used (several answers possible)

- o PCR
- o immunofluorescence
- o enzyme immuno assay
- o culture
- o serology
- o other: …

1.2 Specimen (several answers possible)

- o nasal swab
- o nasopharyngeal swab
- o nasopharyngeal aspirate
- o wash
- o blood
- o other: …

<u>Age.</u>
2.1 Age at moment of death … months (0-59)

2.2 Date of death: month/year (Jan-Dec)/(1995-2015)

<u>Basic patient characteristics.</u>
3.1 Gender

- o male
- o female
- o unknown

3.2 Severe underlying disease (if yes, several answers possible)
yes/no/unknown

- o congenital heart disease
- o chronic lung disease
- o immunodeficiency
- o genetic/chromosomal disease
- o neuromuscular disease
- o neurodevelopmental disease
- o airway abnormality
- o malignancy
- o other: …

3.3 Prematurity yes/no/unknown
< 37 completed weeks of gestation

3.4 Length/height at admission (use cm or in) … centimetre (cm)
Or current length if out of hospital patient … inches (in)

3.5 Weight at admission (use kg or lbs): … kilogram (kg)
Or current weight if out of hospital patient … pounds (lbs)

3.6 Current type of feeding
Current feeding if child is < 4 months of age. If child is > 4 months of age report feeding in first 4 months of life

- o exclusive breast feeding
- o mixed breast & bottle feeding

- exclusive bottle feeding
- unknown
- other: …

<u>Hospital admission.</u>

4.1 Hospitalisation yes/no/unknown

4.2 Length of stay in hospital (if applicable) … days

4.3 Intensive care admission yes/no/unknown, if yes … days

4.4 Mechanical ventilation yes/no/unknown, if yes … days

4.5 Time interval between onset of RSV related symptoms and hospital admission … days

4.6 Contact with other health care providers before hospitalisation yes/no/unknown

4.7 Place of death; in hospital? yes/no/unknown

4.8 Time interval between onset of RSV related symptoms and death … days

<u>Clinical characteristics.</u>

5.1 Clinical symptoms present (several answers possible):
- cough
- difficult breathing
- fast breathing (age < 2 months, ≥ 60 breaths; age 2–11 months, ≥ 50/min; age 1–5 years, ≥ 40/min)
- chest indrawing
- central cyanosis
- severe respiratory distress (e.g. grunting, very severe chest indrawing)
- inability to breastfeed or drink, vomiting everything
- lethargy, reduced level of consciousness or convulsions

5.2 Co-infection present yes/no/unknown

if y:
- malaria
- HIV/AIDS
- TB
- other: …

5.3 Oxygen saturation (SpO2) on room air upon hospital admission …%

5.4 WBC count
- <6000/cu mm
- 6000-25.000/cu mm
- >25.000/cu mm
- unknown

5.5 Haemoglobin level
- <5 g/dL
- 5-<8 g/dL
- 8-10 g/dL
- >10 g/dL
- unknown

5.6 Other respiratory virus or bacteria present in respiratory sample yes/no/unknown

if yes (several answers possible):

- o Influenza A/B
- o Para-influenza virus
- o Human metapneumovirus
- o Adenovirus
- o Rhinovirus
- o Streptococcus pneumoniae
- o Haemophilus influenzae
- o Mycoplasma pneumoniae
- o other pathogen or sample: …

5.7 Hospital-acquired RSV infection yes/no/unknown

History

6.1 Gestational age … weeks estimated by: LMP/ultrasonography/unknown/both

6.2 Birth weight (use kg or lbs) … kilogram
… pounds

6.3 Immunisation status (specify per vaccine)

Fully means according to local immunization schedule and age. Partially means 1 or more dose(s) missed.

PCV fully/partially/not/unknown
Hib fully/partially/not/unknown
MMR fully/partially/not/unknown
BCG fully/partially/not/unknown
DTP fully/partially/not/unknown

Socio-demographic characteristics.

7.1 RSV seasonality

Estimated start and end of RSV season during calendar year

- o year round (no clear peak season)
- o yearly on average 1 peak season
  start: … end: …
- o yearly on average 2 peak seasons
  start: … end: … of peak season 1
  start: … end: … of peak season 2
- o timing of peak season varies heavily per year
- o unknown
- o other: …

7.2 Other children in household yes/no/unknown

7.3 Day care attendance yes/no/unknown

7.4 Living area rural/urban/unknown

7.5 Maternal education (highest level attended)

- o uneducated
- o primary school level
- o secondary school level
- o university level
- o unknown

7.6 Parental smoking yes/no/unknown

7.7 Comments or additional information
